# Potentially inappropriate medications use in a psychiatric elderly care hospital: A cross‐sectional study using Beers criteria

**DOI:** 10.1002/hsr2.1247

**Published:** 2023-05-23

**Authors:** Gracia Yaghi, Bahia Chahine

**Affiliations:** ^1^ School of Pharmacy Lebanese International University Beirut Lebanon

**Keywords:** Beers criteria, Lebanon, older adults, polypharmacy, potentially inappropriate medication, psychiatric disorders, psychogeriatric unit

## Abstract

**Background and Aims:**

Potentially inappropriate medications (PIMs) carry risks that outweigh any potential benefits when compared to safer or more effective alternative treatments. Adverse drug events are more likely to occur in older adults with psychiatric diseases due to multimorbidity, polypharmacy, and age‐related changes in drug pharmacokinetics and pharmacodynamics. The aim of this study was to assess the prevalence and risk factors of PIM use in an aged care hospital's psychogeriatric division, using the American Geriatrics Society Beers criteria 2019.

**Methods:**

This cross‐sectional study was conducted on all current inpatients, having a mental disorder and aged ≥65 years, in one elderly care hospital in Beirut, from March to May 2022. Medications, sociodemographic and clinical characteristics were collected from patients' medical records. PIMs were evaluated based on Beers criteria 2019. Independent variables were described using descriptive statistics. Factors associated with PIM use were identified by bivariate analysis followed by binary logistic regression. A two‐sided *p* value <0.05 was considered statistically significant.

**Results:**

The study included 147 patients, with a mean age of 76.3 years, 46.9% of them having schizophrenia, 68.7% using 5 or more drugs and 90.5% taking at least 1 PIM. The most prescribed PIMs were antipsychotics (40.2%), anticholinergics (16%), and antidepressants (7.8%). PIM use was significantly associated with polypharmacy (AOR = 20.88, 95% CI: 1.22−357.87, *p* = 0.04) and anticholinergic cognitive burden (ACB) score (AOR = 7.25, 95% CI: 1.13−46.52, *p* = 0.04).

**Conclusion:**

PIMs were highly prevalent among hospitalized Lebanese psychiatric elderly. Polypharmacy and ACB score were the determinants of PIM use. A multidisciplinary medication review led by a clinical pharmacist could reduce PIM use.

## 1 INTRODUCTION

Mental disorders are disturbances in a person's cognition, emotional control, or behavior that cause distress or functional impairment in one or more major life domains. Mental disorders, including dementia and substance use disorders, are very common among older adults, 65 years and over, with a worldwide estimated prevalence of 21.4%, according to Global Burden of Disease Study 2019 results.^1^ In Lebanon, their prevalence is even higher reaching 23.2% of older adults who represent 9.7% of the population.^1^, ^2^

Older adults in general and those with psychiatric illnesses in particular are at greater risk of adverse drug events (ADEs) due to multimorbidity, polypharmacy, and age‐related changes in drug pharmacokinetics and pharmacodynamics, notably increased sensitivity to the central nervous system (CNS) and anticholinergic effects of drugs.^3^ Moreover, older adults with psychiatric illnesses are more exposed^4^ to PIM use which is defined as the intake of drugs with ineffectiveness or a high risk‐benefit ratio, compared with more effective and safer alternative treatment options.^5^ Numerous studies have found polypharmacy, which is commonly reported as the daily use of 5 or more drugs,^6^ to be a predictor of PIM use.^7^, ^8^ The latter is a major health concern associated with increased healthcare costs and poor clinical outcomes, such as hospitalizations, emergency department visits, outpatient provider visits, prolonged length of stay, functional decline, cognitive impairment, many other geriatric syndromes, ADEs, and even mortality.^8^, ^9^, ^10^

Several criteria have been developed in different countries to evaluate the appropriateness of medications use in older adults.^11^ The American Geriatrics Society (AGS) Beers criteria are the most utilized and the longest‐running criteria in all settings of care, except for the hospice and palliative care settings.^12^ The latest AGS Beers criteria update was the 2019 version^12^ In addition to identifying medications for which the risks of use outweigh the benefits, the AGS Beers criteria also highlight the subgroups of older adults who should be exempt from or to whom they apply.^12^ Moreover, they draw attention of clinicians to potential problems and interactions when considering the use of these medications.^12^ However, they are only a starting point for improving medication appropriateness since they don't cover underuse and overuse of drugs. The AGS recommends clinicians to use clinical judgment and common sense when prescribing medications, knowing that full adherence to the criteria is not always practicable. Nevertheless, the designation of the medication as potentially inappropriate can serve as a reminder for close monitoring if a clinician is unable to find an alternative and decides to continue using a PIM in a specific patient. This will allow the possibility of prevention or early detection of an ADE.

The prevalence of potentially inappropriate medications (PIMs) varied widely depending on the study settings, the patient characteristics, the version of the Beers criteria and the country‐specific market regulations that affect how identical criteria are applied. It ranged from 18.5% to 82.6% in residential long‐term care facilities for older adults,^13^ while it varied from 20.6% to 80.5% among older inpatients when cognitive impairment was reported.^14^ Several studies showed that anxiolytics were the most common PIMs in psychiatric older adults, followed by antidepressants and antipsychotics.^7^

Although several studies in various countries have used different assessment tools to examine the prevalence, predictors, and impact of PIM use in older adults in a range of settings,^15^ few studies assessed the appropriateness of prescribing in psychiatric older adults generally and those staying in nursing homes specifically. Thus, having a better understanding of the prevalence and contributing factors of PIM use, as a whole, among psychiatric older adults living in nursing homes, can help in enhancing clinical outcomes and lowering healthcare costs.

In Lebanon, no studies have been performed on PIM use among psychiatric elderly in chronic residential care. Thereby, the aim of this study was to assess the prevalence and risk factors of PIM use in the psychogeriatric division of an elderly care hospital in Beirut, through the utilization of the AGS Beers criteria 2019.

## 2 METHODS

### 2.1 Study design, setting, and participants

This cross‐sectional, observational, and single‐centered study was conducted from March to May 2022 in a 600‐bed elderly care hospital in Beirut, Lebanon. The geriatrics division of the hospital is committed to optimizing the health of about 300 elderly resident‐patients (long‐stay inpatients) who have multiple chronic conditions, life‐limiting illness, frailty, or disability caused by chronic disease, aging or injury. The hospital also includes a psychogeriatric division that treats 300 resident‐patients with psychiatric or psychological disorders, such as depression, anxiety, bipolar disorder, or schizophrenia. The study sample included all current inpatients, aged 65 years and older, diagnosed with a mental disorder, taking at least one chronic medication and having received a comprehensive geriatric assessment by a trained physician. Patients with incomplete medical records were excluded. The sample size included all patients who met the eligibility criteria during the study period. Therefore, the sample size was not calculated a priori.

The study protocol was approved by the Institutional Review Board (2022RC‐006‐LIUSOP) and was in compliance with the Declaration of Helsinki. The collection and processing of the data in this study complied with data protection and privacy. A written informed consent from the patients or their caregivers was not required since this study was observational in nature and did not have any physiologic, psychological, or social risks on the participants.

### 2.2 Data collection and variables

During a 2‐month visit to the psychogeriatric unit, a researcher conducted a standardized assessment of medical records, including the clinical case notes, for all patients who met the inclusion criteria. The sociodemographic and clinical information was extracted from each patient's hard copy medical record and entered into an electronic data collection form. Comorbidities were reported according to the WHO's International Classification of Diseases, Tenth Revision, Clinical Modification (ICD‐10‐CM).^16^ The list of current medications, including brand and generic name, dosage form, dose, frequency, route of administration, and date of initiation were also recorded. Furthermore, body mass index (BMI)^17^ as well as the estimated glomerular filtration rate and creatinine clearance (CrCl) using the Chronic Kidney Disease Epidemiology Collaboration (CKD‐EPI) equation^18^ were computed.

The level of dependency was evaluated using the Katz index of independence in activities of daily living (ADL) which graded the ability to perform the six activities of bathing, dressing, toileting, transferring, continence, and feeding on a binary scale of independence or dependence. It was divided into two categories: independence or moderate functional impairment for scores of 3−6 and dependence or severe functional impairment for scores of 2 or less.^19^

Cognitive impairment was defined as a diagnosis of dementia, Alzheimer's, mental retardation, or the identification of cognitive problems in the mental status examination of the psychiatric evaluation. Moreover, the most recent mini mental state examination (MMSE) score,^20^ was reported for patients with dementia or Alzheimer's. Within 2 months of data collection, the attending physician performed the test including 30 items. Patients were classified as having severe (MMSE = 0−10), moderate (MMSE = 11−20), mild (MMSE = 21−25), or no cognitive impairment (MMSE = 26−30).^21^ The MMSE was scored as missing if it was not completed for any reason, including a hearing or visual impairment or another disability.

The comorbidities were categorized using the Charlson comorbidity index (CCI)^22^ into three levels of severity: mild (CCI = 2), moderate (CCI = 3−4), and severe (CCI ≥5). Polypharmacy was defined as the concomitant use of at least five drugs.^6^ Medications were classified according to the WHO's Anatomical Therapeutic Chemical Classification System.^23^ All medication entries were reviewed and approved independently by two clinical pharmacists. Antiepileptics, antipsychotics, benzodiazepines, nonbenzodiazepine, benzodiazepine receptor agonist hypnotics, and antidepressants were considered CNS‐active drugs, according to the AGS Beers criteria 2019.^12^ In addition, the anticholinergic burden of medications, prescribed for more than 6 months, was assessed using the 2012 update of the anticholinergic cognitive burden (ACB) scale.^24^ This scale classify drugs with anticholinergic properties according to their adverse cognitive effects. The total ACB score of each patient was calculated by adding the score of each possible (score 1) and definite (score 2 or 3) anticholinergic drug identified. It was then divided into four groups: 0, 1, 2, and ≥3 due to the increased risk of cognitive impairment and mortality with an ACB score ≥3.^25^

Every medication included in the patient's medical record was assessed for PIM use based on the updated 2019 AGS Beers criteria.^12^ The dependent variable was the use of at least one PIM by the psychiatric older adults. The medication was deemed to be PIM if it was listed in any of the five tables included in the Beers criteria 2019: PIMs to avoid in most older adults, PIMs to avoid in certain diseases and syndromes, PIMs to use with caution, clinically important drug−drug interactions to avoid, and PIMs to avoid or have their dose reduced based on levels of kidney function.

### 2.3 Statistical analysis

All data analyses were performed using IBM® SPSS® Statistics software, version 25. Descriptive statistics were used to describe the demographic and clinical characteristics of psychiatric older adults. Categorical variables were reported using numbers and percentages, whereas continuous variables were presented using means (M) and standard deviations (SD). The association of PIM use with each elderly's characteristic was tested using Student's *t*‐test for continuous variables and *χ* ^2^ test or Fisher's exact test or Fisher−Freeman−Halton test for categorical variables. All variables in the bivariate analysis with a *p* value <0.20 were included in the logistic regression model, unless they were strongly correlated. The binary logistic regression allowed the assessment of relationships between PIM use, a dependent variable (PIM use/no PIM use), and each selected independent variable while controlling for the other key potential covariates. A two‐sided *p* value <0.05 was considered statistically significant.

## 3 RESULTS

A total of 147 psychiatric patients met the inclusion criteria. Table 1 summarized the demographic and clinical characteristics of the study elderly. The mean age was 76.3 (±8.7) years and 53.1% of patients were female. The mean length of stay was 143 (±139) months. Forty‐six percent of elderly were single and most of them (62.6%) were not smokers. A total of 72 patients (49%) had severe functional impairment, while 10.2% exhibited severe cognitive impairment with MMSE scores between 0 and 10. However, cognitive impairment affected 81.6% of elderly. The most prevalent mental disorder was schizophrenia (46.9%) and the most common comorbidity was anemia (83.7%). A severe CCI was present in 43.5% of patients. Moreover, the average number of medications taken daily was 6.5 (±3.4) per older adult. Polypharmacy was found in 68.7% of elderly. Eighty percent of patients took at least one CNS‐active drug, and more than half of the participants had an ACB score of 3 or higher. Sixty‐two percent had history of falls or fracture.

**Table 1:** Study elderly's demographic and clinical characteristics.

Characteristics	All elderly (*N* = 147)	
	*N* (%)	*M* (*SD*)
Sex		
Male	69 (46.9)	
Female	78 (53.1)	
Age, year		76.34 (8.67)
Weight, kg		66.91 (15.03)
Height, cm		163.54 (8.11)
BMI, kg/m^2^		25.03 (5.51)
<18.5	13 (8.8)	
18.5−24.9	70 (47.6)	
25−29.9	45 (30.6)	
≥30	4 (12.9)	
Length of hospital stay, m		143.13 (139.48)
Marital status		
Single	68 (46.3)	
Married	13 (8.8)	
Divorced	27 (18.4)	
Widow	39 (26.5)	
Currently smoking	55 (37.4)	
Educational level		
No schooling	55 (37.4)	
High school	79 (53.8)	
University degree	13 (8.8)	
Living		
Alone	35 (23.8)	
With family	112 (76.2)	
Health coverage type		
Private	17 (11.6)	
MOPH	122 (83.0)	
UNRWA	8 (5.4)	
GFR categories		
G1	78 (53.1)	
G2	57 (38.8)	
G3a	8 (5.4)	
G3b	4 (2.7)	
Level of dependency^a^		
Dependent	72 (49.0)	
Independent	75 (51.0)	
MMSE		13.58 (6.34)
0−10	15 (10.2)	
11−20	21 (14.3)	
21−25	7 (4.8)	
Not available/not applicable	104 (70.7)	
Charlson comorbidity index		4.37 (1.60)
2	22 (15.0)	
3−4	61 (41.5)	
5−9	64 (43.5)	
Mental and nervous system disorders		
Schizophrenia	69 (46.9)	
Alzheimer's	34 (23.1)	
Dementia	23 (15.6)	
Insomnia	36 (24.5)	
Mental retardation	24 (16.3)	
Depression	19 (12.9)	
Acute and chronic diseases		
Hypertension	74 (50.3)	
Dyslipidemia	63 (42.9)	
Diabetes mellitus	44 (29.9)	
Anemia	123 (83.7)	
Osteoporosis	29 (19.7)	
Goiter/thyroid disease	24 (16.3)	
COPD	12 (8.2)	
Dermatological diseases	79 (53.7)	
Eosinophilia	54 (36.7)	
UTI	49 (33.3)	
Eye diseases	33 (22.4)	
Circulatory diseases	33 (22.4)	
Musculoskeletal and connective tissue diseases	33 (22.4)	
Digestive diseases	43 (29.3)	
Abnormal laboratory findings	108 (73.5)	
Geriatric syndromes		
Bladder bowel incontinence	86 (58.5)	
History of falls or fracture	91 (61.9)	
Cognitive impairment	120 (81.6)	
Number of medications		6.48 (3.36)
1−4	46 (31.3)	
5−16	101 (68.7)	
Number of CNS‐active drugs		1.34 (1.04)
0	30 (20.4)	
1	62 (42.2)	
2	35 (23.8)	
≥3	20 (13.7)	
Anticholinergic cognitive burden score		3.31 (3.13)
0	30 (20.4)	
1	27 (18.4)	
2	15 (10.2)	
≥3	75 (51.0)	

Abbreviations: BMI, body mass index; CNS, central nervous system; COPD, chronic obstructive pulmonary disease; GFR, glomerular filtration rate; M, mean; MMSE, mini‐mental state exam; MOPH, Ministry of Public Health; PIM, potentially inappropriate medication; SD, standard deviation; UNRWA, United Nations Relief and Works Agency for Palestine refugees; UTI, urinary tract infection.

^a^ Based on KATZ Index of Independence in activities of daily living score.

The study identified 133 psychiatric older adults using at least one PIM based on the AGS Beers criteria 2019. So the prevalence of PIM use was 90.5%. The distribution of PIM users by number of PIMs showed that 31.6% of them received one PIM, 25.6% received 2 PIMs and 42.8% received 3 or more PIMs (Supporting Information: Figure S1‐Appendix).

The majority of PIMs prescribed fell into three main categories: PIMs due to drug−disease or drug−syndrome interactions 274 (41%), PIMs to be used with caution 210 (31.4%) and PIMs to be avoided 130 (19.5%) affecting 73.5%, 81.6%, and 61.2% of elderly, respectively (Table 2).

**Table 2:** PIMs categories according to 2019 AGS Beers criteria.

PIM category	PIM, *N* (%)	All elderly (*N* = 147)	
		Elderly without PIM *N* (%)	Elderly with PIM *N* (%)
PIMs to be avoided	130 (19.5)	57 (38.8)	90 (61.2)
PIMs due to drug−disease or drug−syndrome interactions	274 (41.0)	39 (26.5)	108 (73.5)
PIMs to be used with caution	210 (31.4)	27 (18.4)	120 (81.6)
PIMs due to drug−drug interactions	53 (7.9)	106 (72.1)	41 (27.9)
PIMs based on the kidney function	1 (0.2)	146 (99.3)	1 (0.7)
Total	668 (100.0)	14 (9.5)	133 (90.5)

Abbreviations: AGS, American Geriatrics Society; PIM, potentially inappropriate medication.

As indicated in Table 3, there were 130 PIMs to be avoided according to the Beers criteria 2019. The most often prescribed PIMs in this category were antipsychotics (26.2%), such as risperidone (13.8%), anticholinergics (37.7%), mostly trihexyphenidyl (20.7%) and promethazine (15.4%), PPI (14.6%), mainly omeprazole (13.8%) and benzodiazepines (10%), primarily lorazepam (7.7%).

**Table 3:** PIMs to be avoided according to 2019 AGS Beers criteria.

Organ system, therapeutic category, drugs	PIM, *N* (%)
Anticholinergics	49 (37.7)
Antihistamines, first‐generation	21 (16.2)
Dimenhydrinate	1 (0.8)
Promethazine	20 (15.4)
Antiparkinsonian agents, trihexyphenidyl	27 (20.7)
Antispasmodics, belladonna alkaloids/prochlorperazine/meprobamate	1 (0.8)
Cardiovascular	4 (3.1)
Central alpha‐agonists, moxonidine	1 (0.8)
Cardiac glycosides, digoxin	2 (1.5)
Antiarrhythmics, amiodarone	1 (0.8)
Central nervous system	49 (37.7)
Antidepressants, clomipramine	2 (1.5)
Antipsychotics	33 (25.4)
Chlorpromazine	3 (2.3)
Haloperidol	4 (3.1)
Quetiapine	5 (3.8)
Risperidone	18 (13.8)
Other: flupentixol/melitracen, zuclopenthixol, olanzapine	3 (2.4)
Benzodiazepines	13 (10.0)
Intermediate acting, lorazepam	10 (7.7)
Long Acting: clonazepam, diazepam	3 (2.3)
Nonbenzodiazepine, benzodiazepine receptor agonist hypnotics, eszopiclone	1 (0.8)
Endocrine	9 (6.9)
Insulins, sliding scale	5 (3.8)
Sulfonylureas, long acting	4 (3.1)
Gliclazide	3 (2.3)
Glimepiride	1 (0.8)
Gastrointestinal	19 (14.6)
Proton‐pump inhibitors	19 (14.6)
Omeprazole	18 (13.8)
Esomeprazole	1 (0.8)
Total	130 (100.0)

Abbreviations: AGS, American Geriatrics Society; PIM, potentially inappropriate medication.

A total of 274 drug−disease or drug−syndrome interactions that may exacerbate the disease or syndrome were identified (Table 4). The most common were the use of antipsychotics in patients with dementia or cognitive impairment (35.7%) and in patients with history of falls or fractures (25.8%), followed by the use of anticholinergics in patients with dementia or cognitive impairment (13.9%).

**Table 4:** PIMs due to drug−disease or drug−syndrome interactions according to 2019 AGS Beers criteria.

System, disease, or syndrome	Therapeutic category, drugs	PIM, *N* (%)
Cardiovascular		
Heart failure	Antithrombotic, aspirin	3 (1.1)
Central nervous system		
Dementia or cognitive impairment	Anticholinergics	38 (13.9)
	Promethazine	13 (4.7)
	Trihexyphenidyl	20 (7.3)
	Other: Baclofen, tizanidine, solifenacin, dimenhydrinate	5 (1.9)
	Antipsychotics	98 (35.7)
	Chlorpromazine	21 (7.6)
	Flupentixol/melitracen	1 (0.4)
	Haloperidol	26 (9.5)
	Zuclopenthixol	4 (1.4)
	Clozapine	6 (2.2)
	Olanzapine	3 (1.1)
	Quetiapine	6 (2.2)
	Risperidone	31 (11.3)
	Benzodiazepines	12 (4.4)
	Lorazepam	10 (3.6)
	Other: Clonazepam, diazepam	2 (0.8)
	Nonbenzodiazepine, benzodiazepine receptor agonist hypnotics, eszopiclone	1 (0.4)
History of falls or fractures	Antidepressants	16 (5.8)
	Sertraline	12 (4.3)
	Other: Clomipramine, escitalopram, fluoxetine	4 (1.5)
	Antipsychotics	71 (25.8)
	Chlorpromazine	17 (6.2)
	Haloperidol	14 (5.1)
	Zuclopenthixol	4 (1.4)
	Clozapine	6 (2.2)
	Quetiapine	4 (1.4)
	Risperidone	26 (9.5)
	Benzodiazepines	11 (4.0)
	Lorazepam	9 (3.2)
	Other: Clonazepam, diazepam	2 (0.8)
	Nonbenzodiazepine, benzodiazepine receptor agonist hypnotics, eszopiclone	1 (0.4)
	Antiepileptics	19 (6.9)
	Carbamazepine	4 (1.4)
	Levetiracetam	5 (1.8)
	Topiramate	3 (1.1)
	Valproate	4 (1.4)
	Other: Gabapentin, lacosamide, phenytoin	3 (1.2)
Parkinson disease	Antipsychotics, risperidone	1 (0.4)
Kidney/urinary tract		
Benign prostatic hyperplasia	Anticholinergics: Promethazine, chlorpromazine, olanzapine	3 (1.2)
Total		274 (100)

Abbreviations: AGS, American Geriatrics Society; PIM, potentially inappropriate medication.

In total, 210 PIMs to be used with caution were found with the majority being antipsychotics (63.9%), including risperidone (18.1%), haloperidol (16.2%), and chlorpromazine (15.7%), followed by antidepressants (12.2%), mainly sertraline (9%) and diuretics (11%) like Furosemide (7.6%) (Supporting Information: Table S2‐Appendix). Fifty‐three clinically important drug‐drug interactions to be avoided were detected (Table 5). The concomitant use of 2 or more anticholinergic drugs accounted for 62.3% of these interactions, while the concurrent use of 3 or more CNS‐active drugs accounted for the remaining 37.7%. The only PIM which dosage should be reduced with decreased kidney function was Rivaroxaban with a CrCl of less than 50 mL/min.

**Table 5:** Clinically important drug−drug interactions to be avoided according to 2019 AGS Beers criteria.

Drug−drug interactions	PIM, *N* (%)
≥2 anticholinergics	33 (62.3)
Chlorpromazine−clozapine	3 (5.6)
Chlorpromazine−promethazine	7 (13.2)
Chlorpromazine−trihexyphenidyl	12 (22.6)
Chlorpromazine−promethazine−trihexyphenidyl	2 (3.8)
Chlorpromazine−clozapine−promethazine−trihexyphenidyl	2 (3.8)
Clozapine−trihexyphenidyl	2 (3.8)
Promethazine−trihexyphenidyl	2 (3.8)
Other: Chlorpromazine−clozapine−promethazine/chlorpromazine−clozapine−trihexyphenidyl/clozapine−promethazine	3 (5.6)
≥3 CNS‐active drugs	20 (37.7)
Carbamazepine−chlorpromazine−clozapine−diazepam−haloperidol	1 (1.9)
Carbamazepine−clozapine−haloperidol−sertraline−zuclopenthixol	1 (1.9)
Chlorpromazine−haloperidol−lorazepam−risperidone	1 (1.9)
Chlorpromazine−clozapine−haloperidol	2 (3.8)
Chlorpromazine−clozapine−valproate	2 (3.8)
Chlorpromazine−haloperidol−lorazepam	3 (5.6)
Chlorpromazine−haloperidol−levetiracetam	1 (1.9)
Chlorpromazine−haloperidol−risperidone	1 (1.9)
Chlorpromazine−lorazepam−risperidone	1 (1.9)
Clonazepam−clozapine−zuclopenthixol	1 (1.9)
Clonazepam−flupentixol/melitracen−sertraline	1 (1.9)
Clozapine−haloperidol−zuclopenthixol	1 (1.9)
Escitalopram−haloperidol−quetiapine	1 (1.9)
Haloperidol−levetiracetam−lorazepam	1 (1.9)
Lorazepam−risperidone−valproate	1 (1.9)
Quetiapine−sertraline−valproate	1 (1.9)
Total	53 (100.0)

Abbreviations: AGS, American Geriatrics Society; CNS, central nervous system; PIM, potentially inappropriate medication.

The results of the bivariate analysis showed that PIM users and non‐PIM users differed significantly in terms of gender (*p* = 0.04), age (*p* = 0.002), schizophrenia (*p* = 0.01), Alzheimer's (*p* = 0.004), hypertension (*p* = 0.02), dermatological diseases (*p* = 0.05), bladder bowel incontinence (*p* = 0.03), number of medications (*p* = 0.001), number of CNS‐active drugs (*p* < 0.001), and ACB score (*p* < 0.001) (Table 6 and Supporting Information: Table S5‐Appendix). The binary logistic regression showed that elderly who were taking 5 or more medications were 20.88 times (AOR = 20.88, 95% CI: 1.22−357.87, *p* = 0.04) more likely to be using PIM than those who were taking less than 5 drugs, while 1‐unit increase in ACB score increased odds of PIM use 7.25 times (AOR = 7.25, 95% CI: 1.13−46.52, *p* = 0.04) (Table 6 and Supporting Information: Table S5‐Appendix).

**Table 6:** Bivariate and multivariable analysis of demographic and clinical characteristics associated with PIM use according to 2019 AGS Beers criteria.

Characteristics	PIM use (*N* = 147)		*p* Value	PIM use (*N* = 147)			*p* Value
	No (*n* = 14)	Yes (*n* = 133)		AOR^a^ *R* ^2^ = 0.706^b^	95% CI		
	*N* (%)/*M* (*SD*)	*N* (%)/*M* (*SD*)			Lower AOR	Upper AOR	
Gender							
Male	3 (4.3)	66 (95.7)	0.04^c^	1.408	0.127	15.652	0.78
Female	11 (14.1)	67 (85.9)					
Age, year	83.14 (11.25)	75.62 (8.08)	0.002^d^	0.934	0.817	1.069	0.32
MMSE	10.43 (8.22)	14.19 (5.86)	0.15^d^				0.92
0−10	4 (26.7)	11 (73.3)	0.07^e^				
11−20 (reference: –0 to 10)	2 (9.5)	19 (90.5)		3.444	0.104	113.860	0.49
21−23 (reference: –0 to 10)	1 (14.3)	6 (85.7)		1.541	0.015	161.610	0.85
Schizophrenia	2 (2.9)	67 (97.1)	0.01^c^	0.778	0.031	19.866	0.88
Alzheimer's	8 (23.5)	26 (76.5)	0.004^f^	1.491	0.079	28.219	0.79
Hypertension	3 (4.1)	71 (95.9)	0.02^c^				
Dermatological diseases	11 (13.9)	68 (86.1)	**0.05** c	0.040	0.001	1.109	0.06
Eosinophilia	8 (14.8)	46 (85.2)	0.10^c^	2.183	0.196	24.323	0.53
Bladder bowel incontinence	12 (14.0)	74 (86.0)	0.03^c^	0.297	0.020	4.358	0.38
Number of medications	3.93 (2.62)	6.74 (3.33)	**0.003** d				
1−4	10 (21.7)	36 (78.3)	**0.001** f				
5−16 (polypharmacy)	4 (4.0)	97 (96.0)		20.880	1.218	357.866	0.04
Number of CNS‐active drugs	0 (0.00)	1.48 (0.99)	**<0.001** d				
0	14 (46.7)	16 (53.3)	**<0.001** e				
1	0 (0.0)	62 (100.0)					
2	0 (0.0)	35 (100.0)					
≥3	0 (0.0)	20 (100.0)					
Anticholinergic burden score	0.29 (0.73)	3.62 (3.12)	**<0.001** d	7.254	1.131	46.521	0.04
0	12 (40.0)	18 (60.0)	**<0.001** e				
1	0 (0.0)	27 (100.0)					
2	2 (13.3)	13 (86.7)					
≥3	0 (0.0)	75 (100.0)					

*Note*: Bold values are statistically significant.

Abbreviations: AGS, American Geriatrics Society Beers criteria; M, mean; MMSE, mini‐mental state exam; PIM, potentially inappropriate medication; SD, standard deviation.

^a^ AOR: adjusted odds ratio for all the variables.

^b^ *R* ^2^: Nagelkerke *R*.^2^

^c^ *χ* ^2^ test.

^d^ Independent samples *t*‐test.

^e^ Fisher−Freeman−Halton test.

^f^ Fisher's exact test.

## 4 DISCUSSION

To the best of our knowledge, this is the first study to evaluate prevalence and risk factors of PIM use in Lebanese hospitalized psychiatric elderly.

In this study, the overall prevalence of PIMs among patients with mental disorders was 90.5%, which was higher than the range of 20.6%−82.6% found in earlier studies that used Beers criteria, in similar settings and participant profiles.^14^, ^26^, ^27^, ^28^, ^29^ This could be explained by the inclusion of all PIM categories, as classified by Beers criteria 2019, and the thorough evaluation of all prescribed drugs, not only psychotropic drugs. In fact, if we only considered PIMs to be avoided, PIMs prevalence would be 61.2%, which is less than the 81.4% PIMs prevalence observed in a study analyzing only PIMs to be avoided.^28^ Other studies^29^, ^30^ reported lower PIMs prevalence, ranging from 47% to 82.6%, using Beers criteria 2012, which excluded PIMs based on drug−drug interactions and kidney function. Another study assessing psychotropic PIMs found a reduced prevalence of 35.7% since it only included PIMs to be avoided and PIMs due to drug−drug interactions.^31^ However, when all Beers criteria 2019 were considered, a study's findings matched ours with an overall prevalence of psychotropic PIM use of 91.2%.^32^ Thereby, the vast range of PIMs prevalence may be a result of different study methodologies and Beers criteria versions.

The majority of drugs classified as PIMs in the current study were antipsychotics accounting for 40.2% of overall PIMs. This outcome is consistent with previous studies involving older adults with psychiatric disorders.^26^, ^31^, ^33^ Risperidone (11.2%) and haloperidol (10.3%) were precisely the most prevalent PIMs, similarly to another study of psychiatric outpatients.^27^ Given that 81.6% of elderly had cognitive impairment, antipsychotics represented 35.7% of PIMs to be avoided in patients with dementia or cognitive impairment, due to an increased risk of cerebrovascular accident, cognitive decline, and mortality. They also constituted 25.8% of PIMs to be avoided in patients with history of falls or fractures, since they could exacerbate those conditions that affected 61.9% of elderly. In addition, antipsychotics contributed to 63.9% of PIMs to be used with caution because of the higher risk of hyponatremia, which in fact occurred in 27.3% of older adults.

Anticholinergics were the second‐most commonly given PIMs, accounting for 16% of all PIMs. As listed in Supporting Information: Table S1‐Appendix, they included first‐generation antihistamines, mostly promethazine, antiparkinsonian agents, primarily trihexyphenidyl, antispasmodics in combination with psycholeptics, skeletal muscle relaxants, and antimuscarinics. This prevalence was lower than the 35.7% prevalence found in a study of patients with mild cognitive impairment.^34^ However, it was higher than the range of 1.7%−6% recorded in studies of psychiatric patients.^26^, ^31^ Furthermore, 51% of the patients had an ACB score of at least 3 while only 36.7% of dementia patients had this score.^35^ These disparities may be explained by different prescribing habits, cost of medications and level of physicians' awareness of PIMs. In our study, trihexyphenidyl was used in conjunction with antipsychotics to avoid or treat extrapyramidal symptoms. While promethazine, was primarily prescribed to sedate and treat allergic reactions. The most frequent side effects, constipation (34.5%) and urine retention (20.9%), were indicative of anticholinergics prevalence. Moreover, the concomitant use of 2 or more anticholinergic drugs accounted for 62.3% of drug−drug interactions to be avoided due to cognitive decline‐related risk. Future research could look for the factors associated with higher exposure to anticholinergic drugs in older adults with psychiatric illnesses.

The third most prescribed PIMs were antidepressants, notably the selective serotonin reuptake inhibitor sertraline. They contributed to 7.8% of all PIMs. However, another study^7^ found a substantially higher antidepressants prevalence of 28.4% with a predominance of fluoxetine and amitriptyline. The highly anticholinergic tricyclic antidepressants were also the most used PIMs in a study of psychiatric elderly,^33^ with amitriptyline being prescribed to 37% of them, although selective serotonin reuptake inhibitors are the preferred agents for treating depression.

Thereby, 13.7% of psychiatric elderly were taking 3 or more CNS‐active drugs, which accounted for 37.7% of drug−drug interactions to be avoided, due to a higher risk of falls and fractures. This result was in line with a study conducted on older adults with dementia.^36^

In contrast to our findings, a study found that anxiolytics and hypnotics were the most commonly prescribed PIMs, with a prevalence of 69.9% and 17.2%, respectively.^37^ In another study,^32^ clonazepam, a long‐acting benzodiazepine, was the major PIM. In the current study, benzodiazepines represented 10% of PIMs to be avoided, due to increased risk of cognitive impairment, falls and fractures, with a predominance of Lorazepam, an intermediate‐acting benzodiazepine. The primary cause of this discrepancy could be the shortage of drugs that restricted the use of benzodiazepines while it was perceived as prudent prescribing in a study reporting also a low prevalence of benzodiazepines.^33^

The present study tested the association between PIM use and different demographic and clinical characteristics of psychiatric older adults. In accordance with several studies, polypharmacy or the use of 5 or more drugs was identified as an independent risk factor for PIM use.^7^, ^32^ In fact, 68.7% of older adults had polypharmacy. Increasing the number of medications increases the risk of ADEs and PIM through drug–drug and drug–disease interactions,^38^ especially that approximately half of the participants had a severe CCI. Additionally, more medications are required for patients with more severe psychiatric illnesses; for instance, a meaningful association between having schizophrenia and PIM use was noticed before controlling for potential covariates, while nearly half of the study population had schizophrenia. Furthermore, ADEs can be misdiagnosed as new diseases requiring the addition of potentially avoidable medications, leading to the prescribing cascades.^39^ Thus, a cautious prescribing approach is required to prevent higher healthcare expenditures and several geriatric syndromes, such as cognitive and functional impairment, linked often with inappropriate polypharmacy.^40^

A novel finding from the current study was the significant association between PIM use and an increasing ACB score. Previous studies^41^, ^42^ examined the relationship between ACB score and dementia, cognitive impairment, delirium, or risk of falling but none of these studies showed that ACB score was a predictor of PIM use. Therefore, physicians should try to prescribe safer, equally effective alternatives that have lower anticholinergic activity.

Regarding the association between PIM use and other patient‐related factors like age, gender, and comorbidities, contradictory results have been found. While some studies showed that these characteristics and PIM use were positively associated, other studies revealed no significant association or even a negative correlation.^33^, ^43^, ^44^ None of these factors were found to be significantly associated with PIM use, after adjusting for all potential confounders, in the present study. These discrepancies may be caused by differences in prescribing patterns for frail older adults, physicians' awareness of PIMs, and the influence of other confounding factors.

This study was the first to evaluate all PIM categories of Beers criteria 2019 and to identify determinants of PIM use in hospitalized psychiatric older adults in Lebanon. A complete documentation of all conditions and comorbidities in medical records allowed us to identify PIMs that may exacerbate a disease or syndrome which indeed constituted the major part of PIMs categories. Moreover, it was possible to check for associations between PIM use and multiple indexes of older adults such as CCI, MMSE, KATZ index of independence in ADL, and BMI. Despite this, the study had some limitations. First, it was conducted in one center although it was one of the largest elderly care hospital in Lebanon, offering psychiatric beds. Second, only hospitalized patients were included so extrapolation of results to community‐dwelling older adults may not be feasible. Third, Beers criteria didn't address overuse and underuse of medications. Finally, the cross‐sectional design of the study just revealed associations between variables rather than providing evidence of cause and effect.

## 5 CONCLUSION

This study showed that PIM use, as identified by Beers criteria 2019, was highly prevalent among psychiatric older adults. It also revealed that PIM use was associated with polypharmacy and ACB score. The involvement of a clinical pharmacist as part of a multidisciplinary healthcare team in elderly care hospitals as well as raising physicians' awareness of PIMs lists and increasing the use of nonpharmacological interventions will improve medication appropriateness and lessen drug related problems. Future research should investigate the impact of PIM use on patient‐centered outcomes, such as functional and cognitive status.

## AUTHOR CONTRIBUTIONS

**Gracia Yaghi**: Data curation; formal analysis; software, writing—original draft; writing—review and editing. **Bahia Chahine**: Conceptualization; methodology; supervision; writing—review and editing.

## CONFLICT OF INTEREST STATEMENT

The authors declare no conflict of interest.

## ETHICS STATEMENT

This study was approved by the Institutional review board at the Lebanese International University (approval number 2022RC‐009‐LIUSOP).

## TRANSPARENCY STATEMENT

The lead author Bahia Chahine affirms that this manuscript is an honest, accurate, and transparent account of the study being reported; that no important aspects of the study have been omitted; and that any discrepancies from the study as planned (and, if relevant, registered) have been explained.

## Supporting information

Supporting information.

## Data Availability

The authors confirm that the data supporting the findings of this study are available within the article and its supplementary materials.
