# Supplementary material for: Potentially inappropriate medications use in a psychiatric elderly care hospital: A cross‐sectional study using Beers criteria
Source: Health Sci Rep. 2023 May 23;6(5):e1247. doi: 10.1002/hsr2.1247 (PMC10206279; doi:10.1002/hsr2.1247)
Supplement: Supplementary file 1 — Supporting information. [file HSR2-6-e1247-s001.docx]

Appendix:

**Figure S1.** Distribution of Potentially Inappropriate Medication (PIM) Users (N=133) by Number of PIM


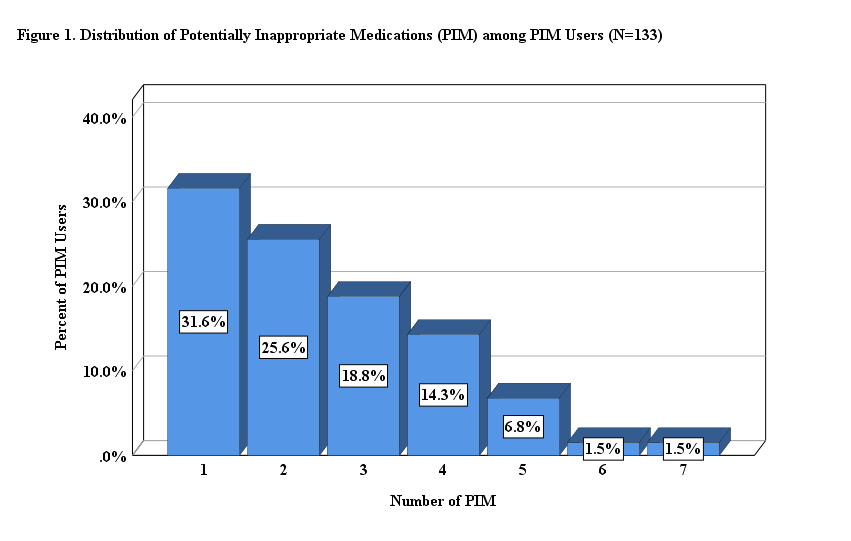


**Table S1.** Overall PIMs According to 2019 AGS Beers Criteria and their ATC Code

| **Organ System, Therapeutic Category, Drugs** | **ATC Code** | **PIM, No. (%)** |
| --- | --- | --- |
| **Anticholinergics** |  | **53 (16.0)** |
| Antihistamines, first-generation |  | 21 (6.3) |
| Dimenhydrinate | R06AA11 | 1 (.3) |
| Promethazine | R06AD02 | 20 (6.0) |
| Antiparkinsonian agents |  | 27 (8.2) |
| Trihexyphenidyl | N04AA01 | 27 (8.2) |
| Antispasmodics in combination with psycholeptics |  | 1 (.3) |
| Belladonna alkaloids/Prochlorperazine/Meprobamate | A03CB02 | 1 (.3) |
| Skeletal muscle relaxants |  | 3 (.9) |
| Baclofen | M03BX01 | 2 (.6) |
| Tizanidine | M03BX02 | 1 (.3) |
| Antimuscarinics, urinary incontinence |  | 1 (.3) |
| Solifenacin | G04BD08 | 1 (.3) |
| **Cardiovascular** |  | **51 (15.3)** |
| Central Alpha-Agonists |  | 1 (.3) |
| Moxonidine | C02AC05 | 1 (.3) |
| Cardiac Glycosides |  | 2 (.6) |
| Digoxin | C01AA05 | 2 (.6) |
| Antiarrhythmics |  | 1 (.3) |
| Amiodarone | C01BD01 | 1 (.3) |
| Antithrombotics |  | 24 (7.2) |
| Aspirin | B01AC06 | 19 (5.7) |
| Rivaroxaban | B01AF01 | 5 (1.5) |
| Diuretics |  | 23 (6.9) |
| Furosemide | C03CA01 | 16 (4.8) |
| Indapamide | C03BA11 | 1 (.3) |
| Hydrochlorothiazide | C03AA03 | 5 (1.5) |
| Spironolactone | C03DA01 | 1 (.3) |
| **Central Nervous System** |  | **199 (60.0)** |
| Antidepressants |  | 26 (7.8) |
| Clomipramine | N06AA04 | 2 (.6) |
| Escitalopram | N06AB10 | 2 (.6) |
| Fluoxetine | N06AB03 | 1 (.3) |
| Sertraline | N06AB06 | 19 (5.7) |
| Mirtazapine | N06AX11 | 2 (.6) |
| Antipsychotics |  | 133 (40.2) |
| Chlorpromazine | N05AA01 | 33 (10.0) |
| Flupentixol/Melitracen | N06CA02 | 1 (.3) |
| Haloperidol | N05AD01 | 34 (10.3) |
| Zuclopenthixol | N05AF05 | 5 (1.5) |
| Amisulpride | N05AL05 | 1 (.3) |
| Clozapine | N05AH02 | 12 (3.6) |
| Olanzapine | N05AH03 | 4 (1.2) |
| Quetiapine | N05AH04 | 6 (1.8) |
| Risperidone | N05AX08 | 37 (11.2) |
| Benzodiazepines |  | 16 (4.8) |
| *Intermediate acting* |  |  |
| Lorazepam | N05BA06 | 12 (3.6) |
| *Long acting* |  |  |
| Clonazepam | N03AE01 | 2 (.6) |
| Diazepam | N05BA01 | 2 (.6) |
| Nonbenzodiazepine, benzodiazepine receptor agonist hypnotics |  | 1 (.3) |
| Eszopiclone | N05CF04 | 1 (.3) |
| Antiepileptics |  | 23 (6.9) |
| Carbamazepine | N03AF01 | 5 (1.5) |
| Gabapentin | N03AX12 | 1 (.3) |
| Lacosamide | N03AX18 | 1 (.3) |
| Levetiracetam | N03AX14 | 6 (1.8) |
| Phenytoin | N03AB02 | 1 (.3) |
| Topiramate | N03AX11 | 3 (.9) |
| Valproic acid | N03AG01 | 6 (1.8) |
| **Endocrine** |  | **9 (2.7)** |
| Insulins |  | 5 (1.5) |
| Insulin, fast acting | A10AB01 | 5 (1.5) |
| Sulfonylureas, long acting |  | 4 (1.2) |
| Gliclazide | A10BB09 | 3 (.9) |
| Glimepiride | A10BB12 | 1 (.3) |
| **Gastrointestinal** |  | **20 (6.0)** |
| Proton Pump Inhibitors |  | 20 (6.0) |
| Omeprazole | A02BC01 | 19 (5.7) |
| Esomeprazole | A02BC05 | 1 (.3) |
| **Total** |  | **332 (100.0)** |
| Abbreviations: PIM, Potentially Inappropriate Medication; AGS, American Geriatrics Society; ATC, Anatomical Therapeutic Chemical; No., number; %, percentage. | | |

**Table S2.** PIMs to Be Used with Caution According to 2019 AGS Beers Criteria

| **Therapeutic Category, Drugs** | **PIM, No. (%)** |
| --- | --- |
| **Antithrombotic** | **22 (10.5)** |
| Aspirin | 17 (8.1) |
| Rivaroxaban | 5 (2.4) |
| **Antidepressants** | **26 (12.2)** |
| Clomipramine | 2 (.9) |
| Escitalopram | 2 (.9) |
| Fluoxetine | 1 (.5) |
| Mirtazapine | 2 (.9) |
| Sertraline | 19 (9.0) |
| **Antipsychotics** | **134 (63.9)** |
| Chlorpromazine | 33 (15.7) |
| Flupentixol/Melitracen | 1 (.5) |
| Haloperidol | 34 (16.2) |
| Zuclopenthixol | 5 (2.4) |
| Amisulpride | 1 (.5) |
| Clozapine | 12 (5.7) |
| Olanzapine | 4 (1.9) |
| Quetiapine | 6 (2.9) |
| Risperidone | 38 (18.1) |
| **Antiepileptics** | **5 (2.4)** |
| Carbamazepine | 5 (2.4) |
| **Diuretics** | **23 (11.0)** |
| Furosemide | 16 (7.6) |
| Hydrochlorothiazide | 5 (2.4) |
| Other: Spironolactone, indapamide | 2 (1.0) |
| **Total** | **210 (100.0)** |
| Abbreviations: PIM, Potentially Inappropriate Medication; AGS, American Geriatrics Society; No., number; %, percentage. | |

**Table S3.** Drugs with Strong Anticholinergic Properties According to 2019 AGS Beers Criteria

| **Therapeutic Category, Drugs** | **PIM, No. (%)** |
| --- | --- |
| **First generation antihistamines,** dimenhydrinate | **1 (1.0)** |
| **Antiemetics,** promethazine | **20 (19.2)** |
| **Antiparkinsonian,** trihexyphenidyl | **27 (26.0)** |
| **Antispasmodics/Antiemetics,** Belladonna alkaloids/Prochlorperazine/Meprobamate | **1 (1.0)** |
| **Skeletal muscle relaxants:** Baclofen, tizanidine | **3 (2.9)** |
| **Antimuscarinics, urinary incontinence,** solifenacin | **1 (1.0)** |
| **Antidepressants,** clomipramine | **2 (1.9)** |
| **Antipsychotics** | **49 (47.0)** |
| Chlorpromazine | 33 (31.7) |
| Clozapine | 12 (11.5) |
| Olanzapine | 4 (3.8) |
| **Total** | **104 (100.0)** |
| Abbreviations: AGS, American Geriatrics Society; PIM, Potentially Inappropriate Medication; No., number; %, percentage. | |

**Table S4.** Side Effects Due to PIMs According to 2019 AGS Beers Criteria

| **Side Effects** | **No. (%)** |
| --- | --- |
| Hyponatremia | 38 (27.3) |
| Orthostatic hypotension | 16 (11.5) |
| Constipation | 48 (34.5) |
| Urinary retention | 29 (20.9) |
| Hypoglycemia | 8 (5.8) |
| **Total** | **139 (100.0)** |
| Abbreviations: PIM, Potentially Inappropriate Medication; AGS, American Geriatrics Society; No., number; %, percentage. | |

**Table S5.** Bivariate and Multivariable Analysis of Demographic and Clinical Characteristics Associated with PIM Use According to 2019 AGS Beers Criteria

| **Characteristics** | **PIM Use (N=147)** | | ***p* value** | **PIM Use (N=147)** | | | ***p* value** |
| --- | --- | --- | --- | --- | --- | --- | --- |
|  | **No (n=14)** | **Yes (n=133)** |  | **AOR^a^  R^2^=.706^b^** | **95% CI** | |  |
|  | **No. (%) / *M* (*SD*)** | **No. (%) / *M* (*SD*)** |  |  | **Lower AOR** | **Upper AOR** |  |
| Gender |  |  |  |  |  |  |  |
| Male | 3 (4.3) | 66 (95.7) | **.04^c^** | 1.408 | .127 | 15.652 | .78 |
| Female | 11 (14.1) | 67 (85.9) |  |  |  |  |  |
| Age, y | *83.14 (11.25)* | *75.62 (8.08)* | **.002^d^** | .934 | .817 | 1.069 | .32 |
| BMI, kg/m^2^ | *24.22 (4.80)* | *25.12 (5.59)* | .56^d^ |  |  |  |  |
| <18.5 | 1 (7.7) | 12 (92.3) | .97^e^ |  |  |  |  |
| 18.5 – 24.9 | 7 (10.0) | 63 (90.0) |  |  |  |  |  |
| 25 – 29.9 | 5 (11.1) | 40 (88.9) |  |  |  |  |  |
| ≥30 | 1 (5.3) | 18 (94.7) |  |  |  |  |  |
| Length of hospital stay, m | *117.87* *(161.59)* | *145.79 (137.37)* | .48^d^ |  |  |  |  |
| Marital status |  |  |  |  |  |  |  |
| Single | 8 (11.8) | 60 (88.2) | .77^e^ |  |  |  |  |
| Married | 0 (0.0) | 13 (100.0) |  |  |  |  |  |
| Divorced | 2 (7.4) | 25 (92.6) |  |  |  |  |  |
| Widow | 4 (10.3) | 35 (89.7) |  |  |  |  |  |
| Currently smoking | 4 (7.3) | 51 (92.7) | .47^c^ |  |  |  |  |
| Educational level |  |  |  |  |  |  |  |
| No schooling | 5 (9.1) | 50 (90.9) | .95^c^ |  |  |  |  |
| High school | 8 (10.1) | 71 (89.9) |  |  |  |  |  |
| University degree | 1 (7.7) | 12 (92.3) |  |  |  |  |  |
| Living |  |  |  |  |  |  |  |
| Alone | 4 (11.4) | 31 (88.6) | .74^f^ |  |  |  |  |
| With family | 10 (8.9) | 102 (91.1) |  |  |  |  |  |
| Health coverage type |  |  |  |  |  |  |  |
| Private | 1 (5.9) | 16 (94.1) | .86^e^ |  |  |  |  |
| MOPH | 12 (9.8) | 110 (90.2) |  |  |  |  |  |
| UNRWA | 1 (12.5) | 7 (87.5) |  |  |  |  |  |
| GFR categories |  |  |  |  |  |  |  |
| G1 | 9 (11.5) | 69 (88.5) | .82^e^ |  |  |  |  |
| G2 | 5 (8.8) | 52 (91.2) |  |  |  |  |  |
| G3a | 0 (0.0) | 8 (100.0) |  |  |  |  |  |
| G3b | 0 (0.0) | 4 (100.0) |  |  |  |  |  |
| Level of dependency^g^ |  |  |  |  |  |  |  |
| Dependent | 9 (12.5) | 63 (87.5) | .23^c^ |  |  |  |  |
| Independent | 5 (6.7) | 70 (93.3) |  |  |  |  |  |
| MMSE | *10.43 (8.22)* | *14.19 (5.86)* | .15^d^ |  |  |  | .92 |
| 0 - 10 | 4 (26.7) | 11 (73.3) | .07^e^ |  |  |  |  |
| 11 – 20  (Reference: 0 - 10) | 2 (9.5) | 19 (90.5) |  | 3.444 | .104 | 113.860 | .49 |
| 21 – 23  (Reference: 0 - 10) | 1 (14.3) | 6 (85.7) |  | 1.541 | .015 | 161.610 | .85 |
| Not available/Not applicable | 7 (6.7) | 97 (93.3) |  |  |  |  |  |
| Charlson Comorbidity Index | *4.64 (1.50)* | *4.34 (1.61)* | .50^d^ |  |  |  |  |
| 2 | 2 (9.1) | 20 (90.9) | .22^c^ |  |  |  |  |
| 3 – 4 | 3 (4.9) | 58 (95.1) |  |  |  |  |  |
| 5 – 9 | 9 (14.1) | 55 (85.9) |  |  |  |  |  |
| Mental and nervous system disorders |  |  |  |  |  |  |  |
| Schizophrenia | 2 (2.9) | 67 (97.1) | **.01^c^** | .778 | .031 | 19.866 | .88 |
| Alzheimer’s | 8 (23.5) | 26 (76.5) | **.004^f^** | 1.491 | .079 | 28.219 | .79 |
| Dementia | 2 (8.7) | 21 (91.3) | >.99^f^ |  |  |  |  |
| Insomnia | 3 (8.3) | 33 (91.7) | >.99^f^ |  |  |  |  |
| Mental retardation | 3 (12.5) | 21 (87.5) | .70^f^ |  |  |  |  |
| Depression | 1 (5.3) | 18 (94.7) | .69^f^ |  |  |  |  |
| Acute and Chronic diseases |  |  |  |  |  |  |  |
| Hypertension | 3 (4.1) | 71 (95.9) | **.02^c^** |  |  |  |  |
| Dyslipidemia | 4 (6.3) | 59 (93.7) | .26^c^ |  |  |  |  |
| Diabetes Mellitus | 2 (4.5) | 42 (95.5) | .23^f^ |  |  |  |  |
| Anemia | 14 (11.4) | 108 (88.6) | .13^f^ |  |  |  |  |
| Osteoporosis | 3 (10.3) | 26 (89.7) | >.99^f^ |  |  |  |  |
| Goiter/Thyroid disease | 2 (8.3) | 22 (91.7) | >.99^f^ |  |  |  |  |
| COPD | 0 (0.0) | 12 (100.0) | .61^f^ |  |  |  |  |
| Dermatological diseases | 11 (13.9) | 68 (86.1) | **.05^c^** | .040 | .001 | 1.109 | .06 |
| Eosinophilia | 8 (14.8) | 46 (85.2) | .10^c^ | 2.183 | .196 | 24.323 | .53 |
| UTI | 3 (6.1) | 46 (93.9) | .39^f^ |  |  |  |  |
| Eye diseases | 3 (9.1) | 30 (90.9) | >.99^f^ |  |  |  |  |
| Circulatory diseases | 3 (9.1) | 30 (90.9) | >.99^f^ |  |  |  |  |
| Musculoskeletal and connective tissue diseases | 3 (9.1) | 30 (90.9) | >.99^f^ |  |  |  |  |
| Digestive diseases | 4 (9.3) | 39 (90.7) | >.99^f^ |  |  |  |  |
| Abnormal laboratory findings | 12 (11.1) | 96 (88.9) | .35^f^ |  |  |  |  |
| Geriatric Syndromes |  |  |  |  |  |  |  |
| Bladder Bowel Incontinence | 12 (14.0) | 74 (86.0) | **.03^c^** | .297 | .020 | 4.358 | .38 |
| History of falls or fracture | 8 (8.8) | 83 (91.2) | .70^c^ |  |  |  |  |
| Cognitive impairment | 13 (10.8) | 107 (89.2) | .47^f^ |  |  |  |  |
| Number of medications | *3.93 (2.62)* | *6.74 (3.33)* | .003^d^ |  |  |  |  |
| 1 - 4 | 10 (21.7) | 36 (78.3) | **.001^f^** |  |  |  |  |
| 5 – 16 (Polypharmacy) | 4 (4.0) | 97 (96.0) |  | 20.880 | 1.218 | 357.866 | **.04** |
| Number of CNS-active drugs | *0 (.00)* | *1.48 (.99)* | **<.001^d^** |  |  |  |  |
| 0 | 14 (46.7) | 16 (53.3) | **<.001^e^** |  |  |  |  |
| 1 | 0 (.0) | 62 (100.0) |  |  |  |  |  |
| 2 | 0 (.0) | 35 (100.0) |  |  |  |  |  |
| ≥ 3 | 0 (.0) | 20 (100.0) |  |  |  |  |  |
| Anticholinergic Burden Score | *0.29 (.73)* | *3.62 (3.12)* | **<.001^d^** | 7.254 | 1.131 | 46.521 | **.04** |
| 0 | 12 (40.0) | 18 (60.0) | **<.001^e^** |  |  |  |  |
| 1 | 0 (0.0) | 27 (100.0) |  |  |  |  |  |
| 2 | 2 (13.3) | 13 (86.7) |  |  |  |  |  |
| ≥ 3 | 0 (0.0) | 75 (100.0) |  |  |  |  |  |
| Abbreviations: PIM, Potentially Inappropriate Medication; AGS, American Geriatrics Society Beers Criteria; No., number; %, percentage; *M*, mean; *SD*, standard deviation; BMI, Body Mass Index; MOPH, Ministry of Public Health; UNRWA, United Nations Relief and Works Agency for Palestine refugees; GFR, glomerular filtration rate; MMSE, Mini-Mental State Exam; COPD, Chronic Obstructive Pulmonary Disease; UTI, Urinary Tract Infection.  ^a^AOR: adjusted odds ratio for all the variables.  ^b^R^2^: Nagelkerke R Square. | | | | | | | |
| ^c^Chi-square test. | | | | | | | |
| ^d^Independent samples t-test. | | | | | | | |
| ^e^Fisher-Freeman-Halton test. | | | | | | | |
| ^f^Fisher’s Exact test. | | | | | | | |
| ^g^Based on KATZ Index of Independence in Activities of Daily Living score. | | | | | | | |
